# A Facile and Simple Method for Preparation of Novel High-Efficient Form-Stable Phase Change Materials Using Biomimetic–Synthetic Polydopamine Microspheres as a Matrix for Thermal Energy Storage

**DOI:** 10.3390/polym11091503

**Published:** 2019-09-15

**Authors:** Junkai Gao, Xi Tang, Zhengshou Chen, Han Ding, Yi Liu, Xuebin Li, Yan Chen

**Affiliations:** 1 School of Port and Transportation Engineering, Zhejiang Ocean University, Zhoushan 316022, China; gaojk@zjou.edu.cn (J.G.); 18858384935@163.com (X.T.); dinghan940519@163.com (H.D.); ydb1629@163.com (Y.L.); lxbsgdsg@163.com (X.L.); 2 Department of Naval Architecture and Ocean Engineering, Zhejiang Ocean University, Zhoushan 316022, China

**Keywords:** biomimetic synthesis, polydopamine microsphere, phase change material, polyethylene glycol, thermal energy storage

## Abstract

Polydopamine microspheres (PDAMs), synthesized using a biomimetic method, were used as a matrix for polyethylene glycol (PEG) to develop a novel high-efficient form-stable phase change material (PEG/PDAM) using a simple vacuum impregnation strategy. The PDAMs were first used as a support for the organic phase change materials, and the biomimetic synthesis of the PDAMs had the advantages of easy operation, mild conditions, and environmental friendliness. The characteristics and thermal properties of the PEG/PDAMs were investigated using SEM, FTIR, XRD, TGA, DSC, and XPS, and the results demonstrated that the PEG/PDAMs possessed favourable heat storage capacity, excellent thermal stability, and reliability, and the melting and freezing latent heats of PEG/PDAM-3 reached 133.20 ± 2.50 J/g and 107.55 ± 4.45 J/g, respectively. Therefore, the PEG/PDAMs possess great potential in real-world applications for thermal energy storage. Additionally, the study on the interaction mechanism between the PEG and PDAMs indicated that PEG was immobilized on the surface of PDAMs through hydrogen bonds between the PEG molecules and the PDAMs. Moreover, the PDAMs can also be used as a matrix for other organic materials for the preparation of form-stable phase change materials.

## 1. Introduction

Currently, human society is progressing rapidly on various fronts. Large amounts of energy and resources are needed to satisfy the requirements of social development; however, the majority of these inputs are limited and non-renewable. A serious waste of natural energy and the irrational use of resources have led to the grievous pollution of the environment. Therefore, significant attention has been paid to the development of new energy storage systems and their utilization by a number of researchers. Phase change materials (PCMs) are indispensable in methods and applications for energy conservation because they absorb and release a large amount of latent heat in the course of the phase change process [1,2,3,4,5].

Phase change materials have various merits, such as high energy density, cyclic utilization, excellent thermal stability, etc. [6], and therefore, PCMs can be used in various applications, such as in buildings, temperature-regulating fabrics, solar energy systems [7], electron device heat transfer [8], industrial waste heat utilization, and air conditioning systems [9,10,11]. Based on different chemical compositions, phase change materials can be classified as organic PCMs, inorganic PCMs, and organic–inorganic mixture PCMs. According to previous studies, organic PCMs possess a number of advantages, such as favourable chemical stability, good thermal stability, low cost, inconspicuous supercooling, and no phase separation [12,13]. However organic PCMs have several deficiencies, such as low thermal conductivity and liquid leakage and erosion during melting, which hinder the application of PCMs in many fields [11,14].

To solve the above problems, matrixes, such as multi-walled nanotubes, expanded graphite, mesoporous silica, activated carbon, among others, were introduced as supports for organic PCMs to synthesize shape stabilized PCMs. Liu et al. [15] prepared a novel form-stable phase change material containing meso-porous carbon and n-octadecane, which had excellent phase change behaviour and a fast thermal-response rate. Li et al. [16] synthesized organic montmorillonite (OMMT)/paraffin/grafted multi-walled nanotube (MWNT) composite phase change material, and the thermal conductivity of OMMT/paraffin increased 34% by adding grafted MWNTs. Ramakrishnan et al. [17] reported a novel composite phase change material (paraffin/EPO) synthesized by a vacuum impregnation method, which included paraffin and hydrophobic expanded perlite (EPO), and the paraffin/EPO exhibited good chemical compatibility and thermal stability. Zhang et al. [18] investigated composite PCMs which utilized silica matrices as the support for paraffin for thermal energy storage, and the results showed that the scaffold of silica matrices with large specific surface areas could improve the phase change properties of the paraffin, and this approach was cost-effective and had great practical application potentials.

Inspired by the strong adhesion of proteins secreted by mussels, Messersmith’s [19] group reported a biomimetic method for surface modification using dopamine (DA). Dopamine can be oxidized and self-polymerized in a weak alkaline aqueous solution, forming a polydopamine (PDA) coating with strong adhesion to the surfaces of a wide variety of materials [20]. These properties endow it with a wide range of applications, such as drug delivery, biosensors, adsorption, etc. [21,22,23,24]. Moreover, dopamine can be oxidized and self-polymerized to form polydopamine microspheres (PDAMs) using a biomimetic synthesis method, and PDAMs can be used as excellent adsorbents to remove pollutants in wastewater. For instance, Zhang et al. [20] used polydopamine microspheres as efficient adsorbents for the removal of Cr(VI) in industrial effluent. Khalil and Neda [25] utilized polydopamine microspheres as novel nanobiopolymers for the biosorption of l-cysteine from aqueous solutions. Inspired by the strong adsorption capacity of polydopamine microspheres toward various organic materials, in this study, PDAMs were synthesized and used as carriers of organic phase change material. Moreover, the preparation of PDAMs is relatively easy, has mild conditions, low energy consumption, and is non-toxic [26,27]. Additionally, to the best of our knowledge, there are no studies on form-stable phase change materials using PDAMs as a support thus far.

**Figure 1:**
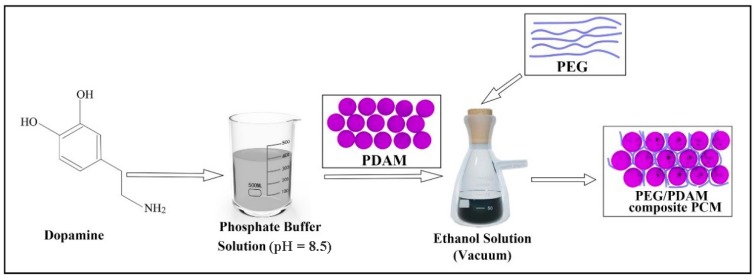
Schematic representation of the preparation process of polyethylene glycol (PEG)/polydopamine microspheres (PDAMs).

Polyethylene glycol (PEG) is a widely used organic phase change material, which has the advantages of a non-toxic nature, high thermal stability, and large latent heat storage [28,29], and therefore, in this work, PDAMs were first synthesized using a biomimetic method, and then they were used to form a matrix of PEG in order to develop a novel and efficient form-stable phase change material (PEG/PDAM) using a simple method of vacuum impregnation. A schematic representation of the preparation process of PEG/PDAMs is shown in Figure 1. The interaction mechanism between PEG and PDAMs was studied, and the properties of PEG/PDAMs, such as phase change behaviour, surface morphology, thermal stability, and reliability were investigated. The results demonstrated that PEG/PDAMs can be used as an efficient energy storage material due to the fact of its high latent heat storage capacity and excellent thermal stability and reliability.

## 2. Experimental Section

### 2.1. Materials

Dopamine hydrochloride was purchased from Sigma-Aldrich (St. Louis, MI, USA). Sodium phosphate dibasic anhydrous and sodium phosphate monobasic anhydrous were purchased from Sinopharm Chemical Reagent Co. LTD (Shanghai, China). Polyethylene glycol was purchased from Aladdin Industrial Corporation (Shanghai, China).

### 2.2. Preparation of PDAMs

The PDAMs were prepared by oxidative polymerization. Phosphate buffer solution with a pH of 8.5 was prepared using hydrogen disodium phosphate and sodium dihydrogen phosphate. Specifically, the phosphate buffer solution (50 mL) and dopamine hydrochloride (0.25 g) were mixed in the beaker (500 mL) to form a homogeneous solution by magnetic stirring (500 rpm) at 25 °C for 24 h. The homogeneous solution was filtered, and then dried for 12 h in an oven at 45 °C to obtain PDAM.

### 2.3. Preparation of PEG/PDAM Composite Materials

Both PEG-4000 and PDAM were used to prepare PEG/PDAM composite materials. Firstly, different concentrations of PEG-4000 solutions were prepared by adding certain amounts of PEG-4000 (0.061 g, 0.093 g, 0.15 g, and 0.20 g) to 10 mL anhydrous ethanol, respectively. Then, a certain quantity of PDAMs (0.05 g, 0.05 g, 0.05 g, and 0.05 g) were blended into the above dissolved solutions, respectively. The mixed solutions were stirred in a vacuum impregnation for 1 h. After that, those mixtures were put in a thermostatic water bath at 45 °C for 4 h. Finally, the mixed solutions were dried at 45 °C for 12 h, and the PEG/PDAM composite materials were acquired, which were then labelled PEG/PDAM-1, PEG/PDAM-2, PEG/PDAM-3, and PEG/PDAM-4, respectively.

### 2.4. Characterization

The size and morphology of the PDAMs and PEG/PDAMs were examined at 10.0 kV by scanning electron microscopy (SEM) with a FEG-250, FEI instrument (Waltham, MA, USA), and the microstructure of the PDAMs was determined by transmission electron microscopy (TEM) with a JEM-2100F, JEOL instrument (Tokyo, Japan). The characteristic functional groups of the PDAMs, PEG/PDAMs, and PEG were ascertained by Fourier transform infrared spectroscopy (FTIR) with a Bruker VECTOR22 system (Karlsruhe, Germany), and the infrared spectra wavenumber range from 500 to 4000 cm^−1^. The crystallization behaviour of the PDAMs, PEG/PDAMs, and PEG were investigated by X-ray diffraction (XRD) with a DX-2700, SHL-2 system from Thermo Scientific (Waltham, MA, USA), which was carried out at 40 kV and 40 mA with a diffraction angle ranging from 10° to 60°. The phase change properties of PEG/PDAMs and PEG were analysed using a differential scanning calorimeter (DSC) with a DSC Q200 system, NETZSCH (Munich, Germany), which aimed to determine the melting and crystallization behaviours of the PEG/PDAMs, and the experiments were carried out from 0 °C to 100 °C with a heating and cooling rate of 10 °C/min under a high purity nitrogen atmosphere. The specific surface areas and pore volumes of the PDAMs were obtained using the Brunauer–Emmett–Teller (BET) method with a Nova 2000 e, Quanta (Waltham, MA, USA) which were carried out at 77.3 K with nitrogen as the analysis gas. The surface chemical compositions of the PDAMs and PEG/PDAMs were measured by X-ray photoelectron spectroscopy (XPS) with an 250Xi K-Alpha system from Thermo Fisher (Waltham, MA, USA). The thermal stability of the PDAMs and PEG/PDAMs were investigated by thermogravimetric analysis (TGA) with a NETZSCH, 209F3 (Selb, Bavaria, Germany), which were conducted from 50 °C to 550 °C with a heating rate of 10 °C/min under a high purity nitrogen atmosphere. For leakage tests, the pure PEG and the composite form-stable PCMs were pressed into small wafers, and these small wafers were placed in an oven at 70 °C for 10 min. If leakage occurred for the PEG/PDAM composites, stains of PEG were left on the filter papers.

## 3. Results and Discussion

### 3.1. Characterization of PDAMs and PEG/PDAMs

**Figure 2:**
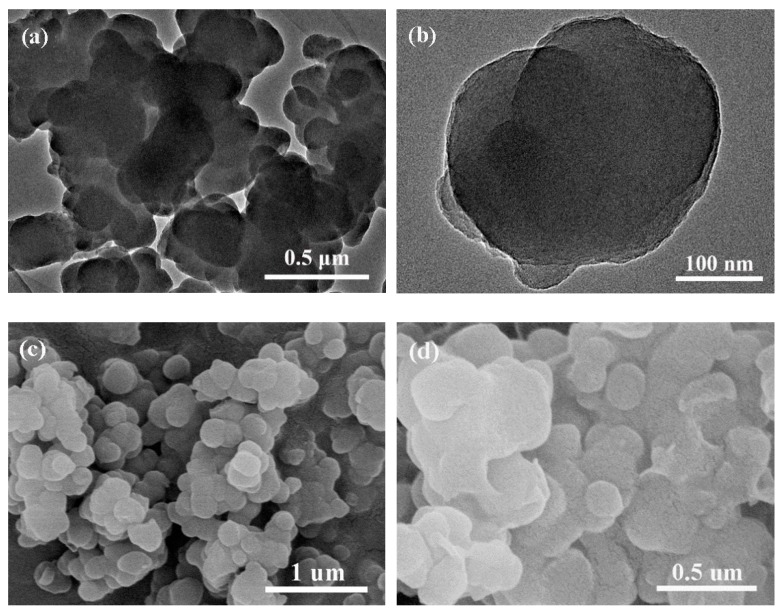

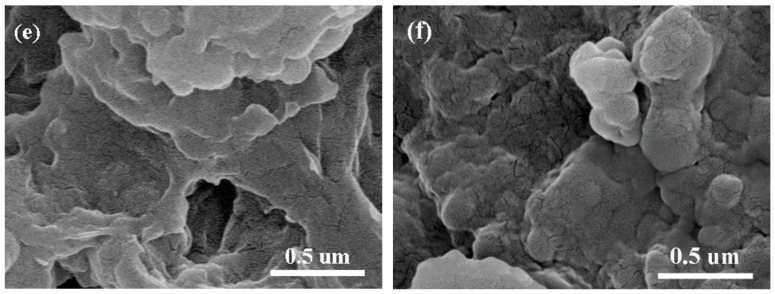
TEM images of PDAMs (**a**,**b**), SEM image of PDAMs (**c**), PEG/PDAM-1 (**d**), PEG/PDAM-2 (**e**), and PEG/PDAM-3 (**f**).

Figure 2 shows the TEM images of the PDAMs and SEM images of the PDAM and PEG/PDAM composites. The TEM images of the PDAMs are shown in Figure 2a,b, which indicated that the PDAMs were a solid structure. Figure 2c shows the SEM image of the PDAMs, and it is clear that the PDAMs had irregular shapes on their outer surface. As is shown in Figure 2d–f, the SEM images of PEG/PDAM-1, PEG/PDAM-2, and PEG/PDAM-3 revealed that the PEG distributed on the surface of all the PEG/PDAM composites, and compared with the images of PEG/PDAM-1 and PEG/PDAM-2, nearly all surfaces of PEG/PDAM-3 were enwrapped with PEG, which might be due to the hydrogen bonds interaction between the PEG molecules and PDAM.

### 3.2. The Nitrogen Adsorption–Desorption Isotherm of the PDAMs

**Figure 3:**
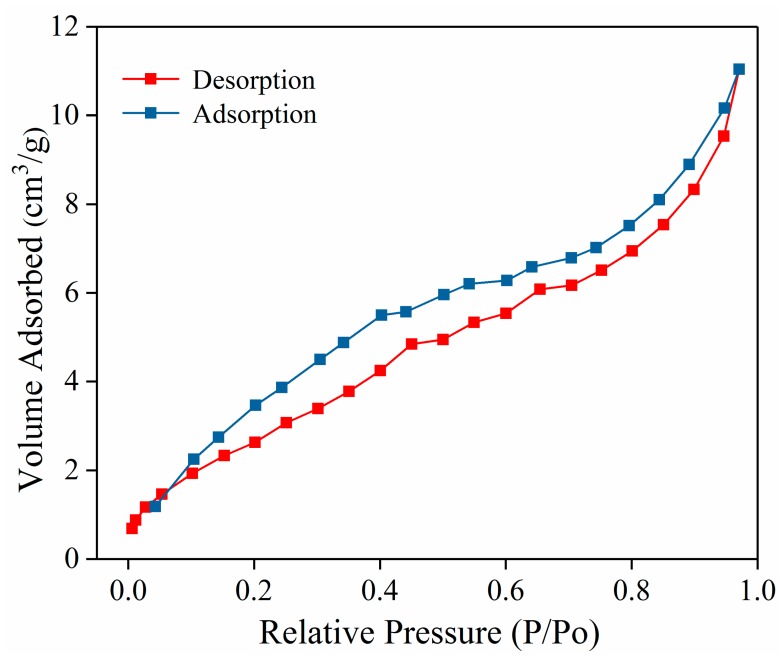
N_2_ adsorption/desorption isotherms of the PDAMs.

Figure 3 shows the nitrogen adsorption–desorption isotherms of the PDAMs obtained at 77.3 K. The surface area of the PDAM nanoparticles was 11.21 ± 0.72 m^2^/g, which was calculated by the BET method. The average pore volume and pore diameter size were calculated to be 0.014 ± 0.0015 cm^3^/g and 3.20 ± 0.18 nm, respectively. Because the PDAMs were a solid structure, the pores originated from the gaps among the polydopamine particles, and the nitrogen adsorption–desorption isotherms of the PDAMs was a type IV isotherm, which indicated that the gaps between the polydopamine particles were mesoporous intervals [30]. More importantly, the large pore diameter of the PDAMs was beneficial for the entrance and accommodation of PEG.

### 3.3. Leakage Tests of PEG and PEG/PDAM Composites

**Figure 4:**
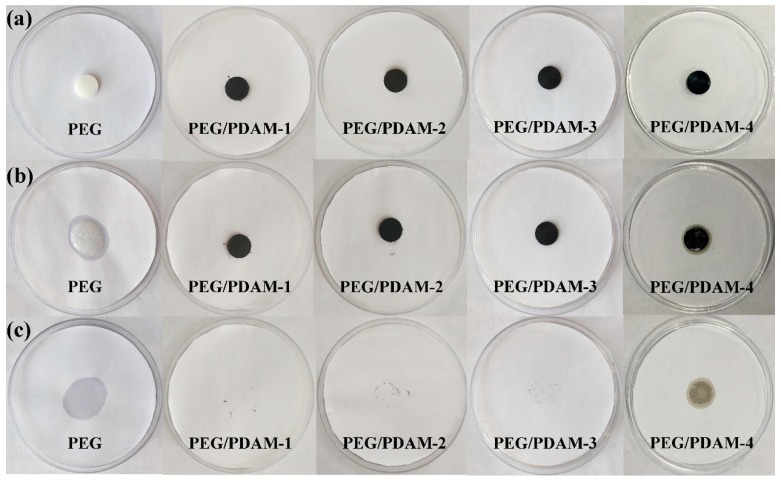
Leakage test photographs of PEG, PEG/PDAM-1, PEG/PDAM-2, PEG/PDAM-3, and PEG/PDAM-4.: (**a**) pictures of samples before the thermal stability test; (**b**) pictures of samples after the thermal stability test; (**c**) pictures of leakage trace after the removal of samples.

Figure 4 shows the leakage test results of pure PEG and PEG/PDAM composites. In the leakage test experiments, if leakage occurred for the PEG/PDAM composites, it left stains of PEG on the filter papers. As shown in Figure 4c, the small wafer of PEG quickly melted into liquid at 70 °C. Meanwhile, the PEG/PDAM-1, PEG/PDAM-2, and PEG/PDAM-3 remained intact and did not show any leakage on the filter paper, which could be attributed to the large adsorption capacity of the PDAMs for the PEG endowing the PEG/PDAM-1, PEG/PDAM-2, and PEG/PDAM-3 with the favourable ability to prevent leakage. However, PEG/PDAM-4 in Figure 4c showed traces of melted PEG, which indicated that liquid leakage occurred for PEG/PDAM-4, and the content of PEG in PEG/PDAM-4 exceeded the load capacity of PDAM. Therefore, on the premise of no leakage appearance, PEG/PDAM-3 could immobilize the largest amount of PEG in this study and, hence, PEG/PDAM-3 served as the main object for researching the thermal properties of the form-stable PCMs.

### 3.4. XRD Patterns of PEG, PDAM, and PEG/PDAM Composites

**Figure 5:**
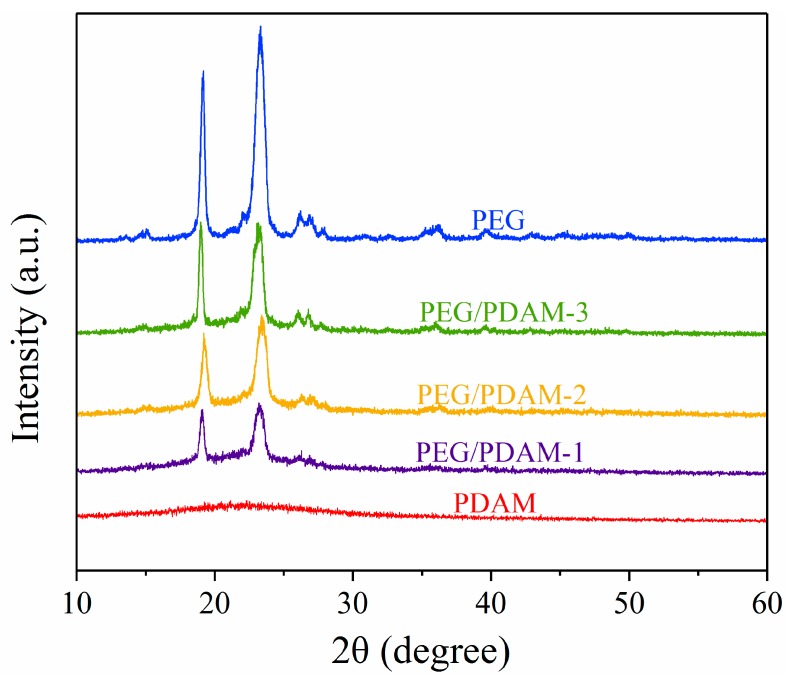
XRD patterns of PEG, PEG/PDAM-1, PEG/PDAM-2, PEG/PDAM-3, and PDAMs.

The XRD patterns of PEG, PDAM, PEG/PDAM-1, PEG/PDAM-2, and PEG/PDAM-3 are shown in Figure 5, where the ordinate values represent the X-ray diffraction intensity and the abscissa values represent the X-ray diffraction angle. The XRD curve of the PDAMs was a flatter curve, and there was no distinct diffraction peak, which indicated that the PDAMs were amorphous [22]. In the XRD curve of pure PEG, there were two sharp and acute diffraction peaks at 19.18° and 23.29°, respectively. The diffraction peak at 19.18° belonged to the {120} plane, and the diffraction peak at 23.29° represents {032} Miller planes of the monoclinic unit cell [31]. The XRD curve of PEG/PDAM-1 appeared at two diffraction peaks at 19.08° and 23.20°, and for the curve of PEG/PDAM-2, there were also two diffraction peaks at 19.20° and 23.40°. Similarly, two typical diffractions peaks at 18.98° and 23.16° could also be observed in the curves of PEG/PDAM-3. It was clear that no new diffraction peak appeared in the XRD curve of the PEG/PDAM composites, and the crystal structure of PEG was almost unchanged in the composite materials of PEG/PDAM-1, PEG/PDAM-2, and PEG/PDAM-3. The latent heat absorption and release of the composites PEG/PDAM was due to the crystallization behaviour of PEG molecules [32], and the crystallization process of PEG included two parts, nucleation of the crystal and growth. In the early period of PEG molecule crystallization, the thermal fluctuations of the polymer chains induced the primary nucleation of the crystal and, subsequently, the stable crystalline phase grew continuously [12]. The diffractions peaks of the XRD curve of the PEG/PDAM composites were significantly lower than that of the pure PEG, which indicated that the interaction between PEG and the PDAMs prevented the crystallization behaviour of PEG molecules [6]. However, it was found that the diffraction peaks of PEG/PDAM-3 was higher than that of PEG/PDAM-1 and PEG/PDAM-2, which demonstrated that with the increase of PEG content in the PEG/PDAM composites, the crystallinity of the composite shape-stabilized phase change materials increased. Furthermore, the results also revealed that the synthesis process of the PEG/PDAM composites was only a physical interaction without any chemical interaction between the PEG molecules and the PDAMs, as there was no change in the crystalline structure of the PEG in the composite phase change materials.

### 3.5. Chemical Properties

**Figure 6:**
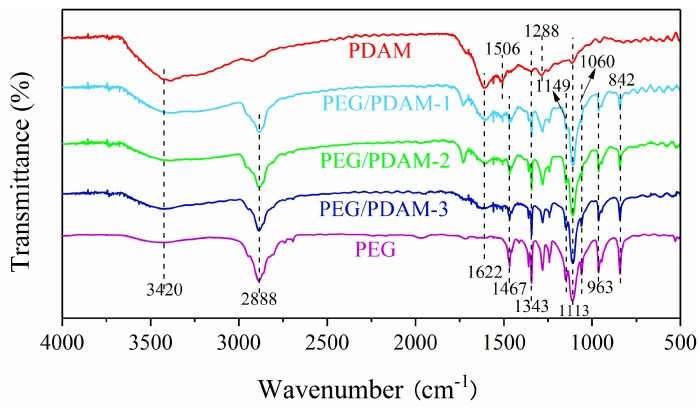
FTIR spectra of PEG, PEG/PDAM-1, PEG/PDAM-2, PEG/PDAM-3, and PDAM.

The FTIR spectra of PEG, PDAMs, PEG/PDAM-1, PEG/PDAM-2, and PEG/PDAM-3 are shown in Figure 6, and in the figure, the ordinate values represent the infrared ray transmittance and the abscissa values represent the infrared ray wavenumber. For the FTIR spectrum of PEG, many narrow peaks were found at 842, 963, 1060, 1113, 1149, 1343, 1467, 1622, 2888, and 3420 cm^−1^, and the peak at 3420 cm^−1^ was attributed to the stretching vibration of the O–H functional group [33]. The peaks at 842, 963, and 2888 cm^−1^ were ascribed to the stretching vibration of –CH2 groups [34], and the peak at 1113 cm^−1^ was attributed to the stretching vibration of the C–O groups [35]. The peak at 1622 cm^−1^ was ascribed to the stretching of water molecules, and the absorption peaks at 1343 cm^−1^ and 1467 cm^−1^ represented the stretching vibration of C–H groups [36]. For the FTIR spectrum of the PDAMs, the adsorption band at 3420 cm^−1^ was considered the stretching vibration of phenolic O–H groups and N–H groups [37]. The peak at 1622 cm^−1^ was considered the stretching vibration of aromatic rings and bending vibration of N–H groups [38,39]. The peaks at 1506 and 1113 cm^−1^ were associated with the N–H shearing vibration of the amide group and the vibration of C–O groups, respectively. The peaks at 1343 and 1288 cm^−1^ could be attributed to the phenolic C–O–H bending and stretching vibration, respectively [6]. The FTIR spectra of PEG/PDAM-1, PEG/PDAM-2, and PEG/PDAM-3 were quite similar to those of PEG and PDAMs, except for some slight variation, which indicated that there was only physical interaction in the synthetic process rather than a chemical reaction.

### 3.6. Latent Heat Storage Analysis

**Figure 7:**
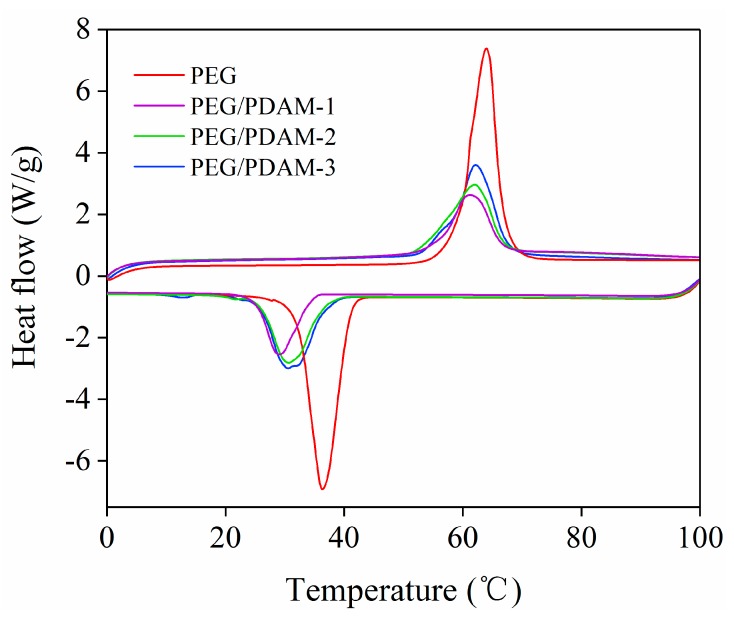
DSC curves of PEG and the samples.

The DSC was used to investigate the melting and crystallizing behaviours of phase change materials. According to the DSC test results, the melting and crystallizing enthalpies of the pure PEG and PEG/PDAM composites are summarized in Table 1. As shown in Figure 7, the DSC curves of the PEG/PDAM composites exhibited a similar shape with that of PEG, meaning that the PEG mainly acted as latent thermal energy storage material during the phase change process. In Table 1, the melting peak temperatures and freezing peak points of the pure PEG were measured as 63.30 ± 0.60 °C and 35.95 ± 0.35 °C, respectively, and the melting and freezing latent heats of PEG reached to 227.40 ± 2.00 J/g and 207.10 ± 0.00 J/g, respectively. Meanwhile, the PEG/PDAM-1 had fusion and solidification enthalpies of 82.57 ± 4.27 J/g and 66.10 ± 4.16 J/g, respectively, and its melting and freezing peak points were 60.95 ± 0.15 °C and 28.05 ± 1.05 °C, respectively. The fusion and solidification enthalpies of PEG/PDAM-2 reached 108.65 ± 1.85 J/g and 91.17 ± 2.44 J/g, respectively, and the melting peak point of PEG/PDAM-2 was 62.00 ± 0.00 °C and its freezing peak point was 29.80 ± 0.80 °C. The fusion and solidification enthalpies of PEG/PDAM-3 reached 133.20 ± 2.50 J/g and 107.55 ± 4.45 J/g, respectively, and its melting and freezing peak points were at 62.20 ± 0.00 °C and 31.10 ± 0.60 °C, respectively. The latent heat value of PEG/PDAM composites with different PEG contents was lower than that of pure PEG, and with increasing PEG contents, the latent heat value of the PEG/PDAM composites were increased.

**Figure 8:**
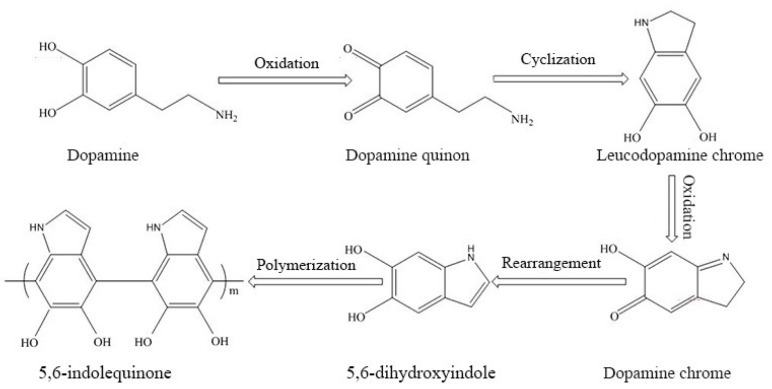
Formation mechanism of polydopamine.

The formation mechanism of polydopamine included four steps, which are shown in Figure 8. Firstly, dopamine formed dopamine quinone easily by oxidation. Secondly, o-dopaminoquinone was formed after the interchange between two electrons and two protons in dopamine molecules in alkaline condition. Quinones were facilely bound with nucleophiles because they have considerably high reactivity, and both the electron-deficient ring and electron-donating amine group were included in the dopaminoquinine [40,41]. Amine groups of dopamine were unprotonated, and cyclization reactions occurring in the molecule were undertaken in 1,4 Michael addition. Then, o-dopaminoquinone generates intramolecular cyclization through 1,4 Michael additions to form leucodopaminochrome. Finally, the leucodopaminochrome was easily oxidized and rearranged, and polydopamine was obtained by reaction and combination between numerous catechols and O-quinone [42].

**Figure 9:**
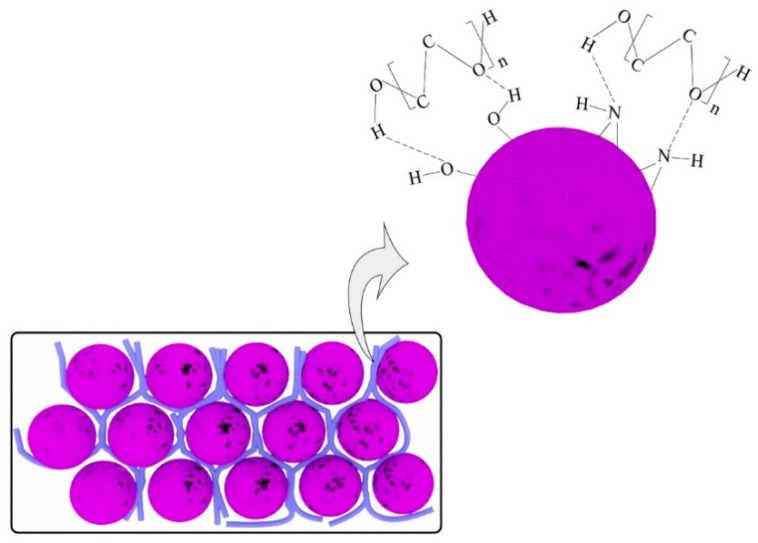
Inferred interaction mechanism between PEG and the PDAMs.

According to the XRD test results, in the preparation process of PEG/PDAM, there was only physical interaction between the PEG molecules and PDAM and, therefore, PEG molecules were immobilized by PDAMs through the capillary force among the gaps of polydopamine microspheres. In addition, according to the FTIR test results, on the surface of PDAMs there were many amino and catechol groups, which can combine with PEG molecules to form hydrogen bonds [43]. The inferred interaction mechanism between PEG and the PDAMs is shown in Figure 9.

**Figure 10:**
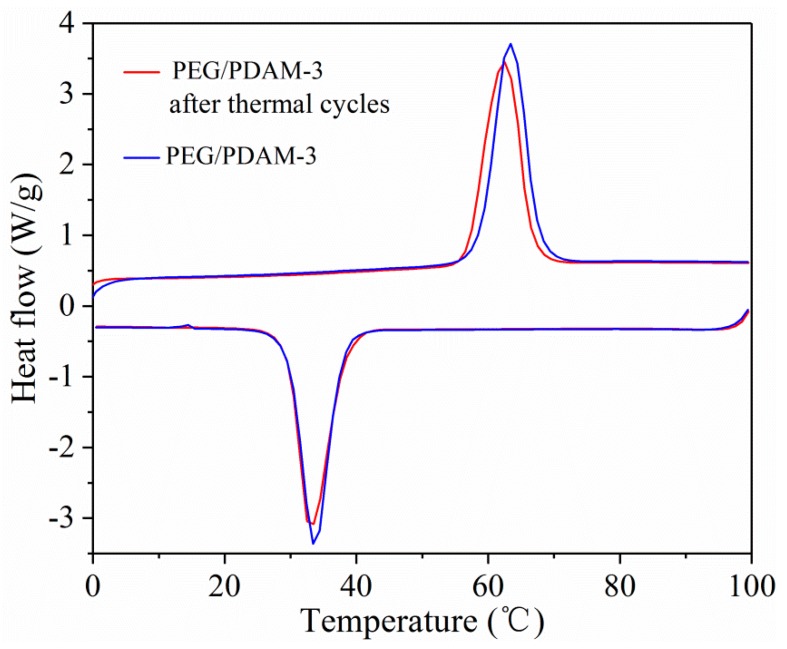
DSC curves of PEG/PDAM-3 before and after the thermal cycle.

In addition to an excellent latent heat storage capacity of a form-stable PCM, it is also very important that form-stable PCMs have perfect thermal reliability. Therefore, thermal cycling tests were carried out to study the thermal reliability of PEG/PDAMs. As shown in Figure 10, the DSC curves of PFG/PDAM-3 before and after 50 thermal cycles changed at a negligible level, demonstrating that, according to the insignificant changes in temperatures and latent heats, PEG/PDAMs possessed excellent thermal reliability and could be repeatedly utilized in the field of thermal energy storage.

### 3.7. Thermal Stability of PEG/PDAM Composites

**Figure 11:**
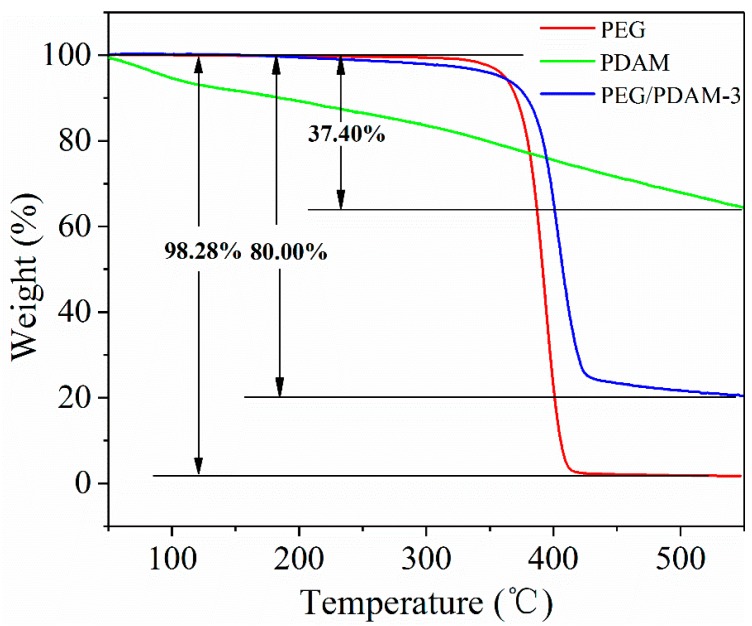
TGA curves of PEG, PDAMs, and PEG/PDAM-3.

Thermal stability is one of the most important parameters of phase change materials for thermal energy storage applications. Figure 11 shows the TGA curves of pure PEG, PDAM, and PEG/PDAM-3. For the sample of pure PEG, there was no distinct change before 310 °C. The TGA curve of pure PEG sharply dropped when the temperature increased from approximately 310 °C to 410 °C, and when the temperature was higher than 470 °C, there was little change, which demonstrated that pure PEG disintegrated completely, with a total weight loss of 98.28%. The TGA curve of the PDAMs is shown in Figure 11, which was deemed to be the typical TGA curve of the PDAMs [32]. It quickly dropped in the temperature range of 50–550 °C, and the total weight loss of the PDAMs was 37.40%. For the sample of PEG/PDAM-3, the TGA curve showed no obvious change when the temperature was below 300 °C. It was observed that the weight of PEG/PDAM-3 decreased rapidly in the temperature range of 300–410 °C. When the temperature was higher than 410 °C, there was a slow change in the weight loss curve of PEG/PDAM-3, and the total weight loss of PEG/PDAM-3 was 80.00%.

According to the results of the TGA tests, the weight loss of pure PEG was 98.28%, with 1.72% of PEG remaining undecomposed, which might be impurities or measurement errors in the tests. The PDAMs exhibited weight losses of 37.40%, and the total weight loss of PEG/PDAM-3 was 80.00%, which contained 10.00% decomposed PDAMs and 70.00% decomposed PEG. Therefore, the content of PEG in the composite of PEG/PDAM-3 was 70.00%. Together with the DSC results of PEG/PDAM-3, the actual latent heat value of PEG/PDAM-3 decreased by 5.00% compared to the theoretical latent heat value of PEG/PDAM-3. This result might be ascribed to the capillary force and van der Waals force between the PDAMs and PEG molecules [43]. The TGA curve of the PDAMs quickly dropped before 200 °C, but there was no obvious change for the TGA curve of PEG/PDAM-3 in the same process. This can be attributed to the content of the PDAMs in the PEG/PDAM-3 being relatively low and, therefore, the weight loss of the PDAMs had little influence on the change in the TGA curve of PEG/PDAM-3 before 200 °C. When the temperature was higher than 410 °C, the curve of PEG/PDAM-3 dropped slowly because the PDAM was still decomposing. Moreover, for the composite of PEG/PDAM-3, the TGA curve did not significantly change before 300 °C, and hence, it is reasonable to believe that PEG/PDAM-3 exhibited favourable thermal stability.

### 3.8. Structural Evolution of PDAMs and PEG/PDAM Composites

**Figure 12:**
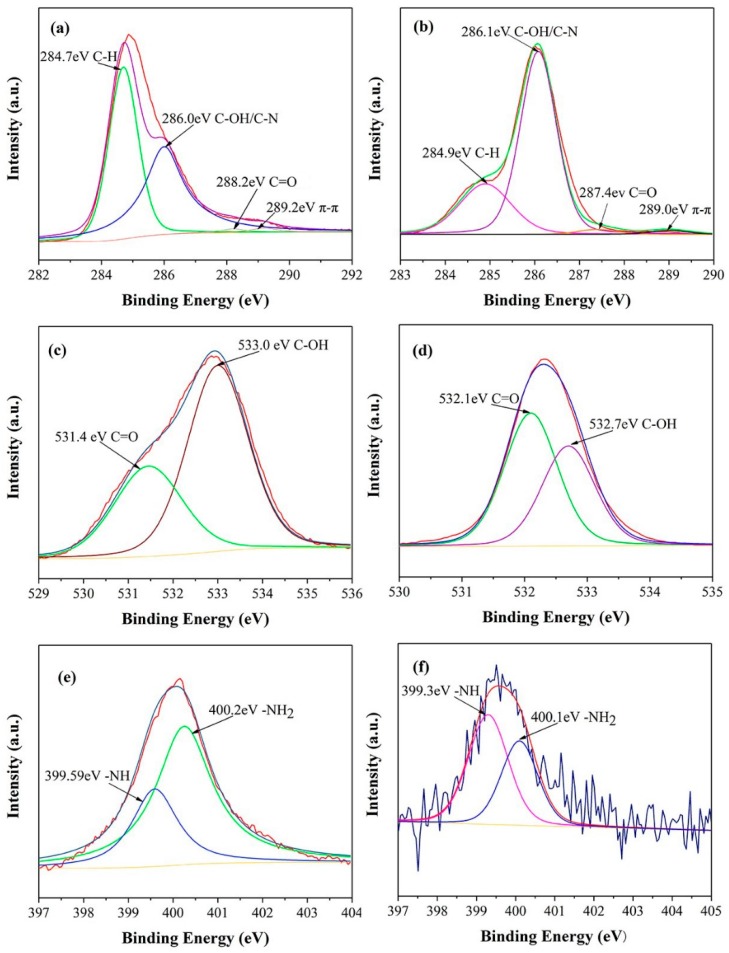
XPS patterns of the PDAMs: (**a**) C, (**c**) O, and (**e**) N, and XPS patterns of 75% PEG/PDAM-3: (**b**) C, (**d**) O, and (**f**) N.

**Figure 13:**
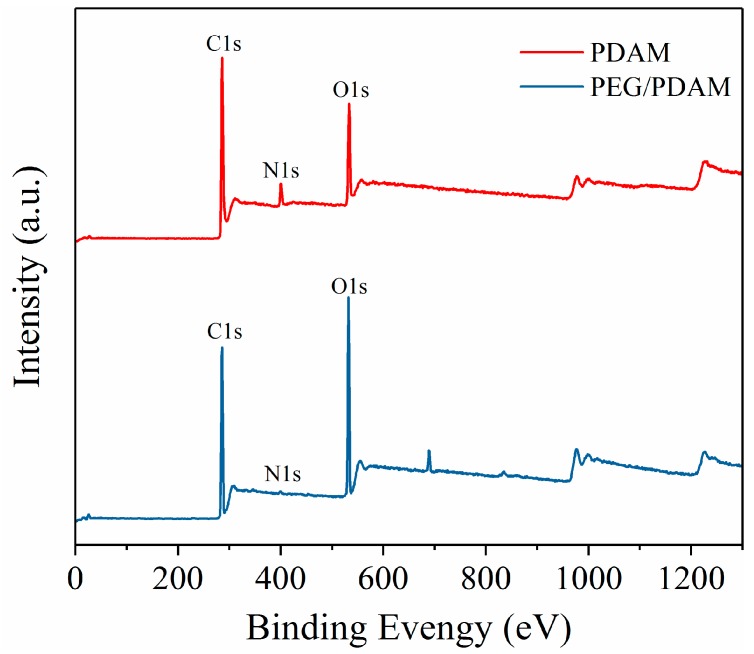
XPS survey of the PDAMs and PEG/PDAM-3.

To further investigate the surface chemical elemental compositions of the PDAMs and PEG/PDAM-3, XPS was carried out to analyse the main elements’ interactions on the surface of the PDAMs and PEG/PDAM-3, and the results are shown in Figure 12, where the ordinate values represent the X-ray photoelectron intensity and the abscissa values represent the electron binding energy. For the XPS curves of the PDAMs, it was obvious that the C1s, N1s, and O1s were the main elements. The peaks of C1s, N1s, and O1s in the XPS curve of PEG/PDAM-3 were located at C–H (284.9 eV), C–OH/C–N (286.1 eV), C=O (287.4 eV), −NH (399.3 eV), −NH2 (400.1 eV), C=O (532.1 eV), and C–OH (532.7 eV), respectively. For the PDAMs, the C1s spectrum had four peak components with aromatic C–H as the most distinct peak at 284.7 eV, and the C–OH/C–N and C=O peaks were located at the 286.0 eV and 288.2 eV, respectively [44,45,46]. Furthermore, the π–π bonding in the aromatic ring was a minor peak at 289.2 eV. Nevertheless, the intensity of π–π bonding in the PEG/PDAM-3 spectrum was decreased, which implied the π–π bonding mutual effect between the PDAM and PEG molecules [47]. Two obvious N1s peaks of PDAM were assigned to −NH2 (400.2 eV) and −NH (399.59 eV), respectively. For the N1s peaks of PEG/PDAM-3, the shifts of the two peaks of −NH2 (400.1 eV) and −NH (399.3 eV) were 0.1 eV and 0.29 eV, respectively, and the N element mainly originated from the ammonium group of the PDAMs, which interacted with the O and H atoms in PEG molecules to form strong hydrogen bonds [48]. For the O1s peaks of the PDAMs, the peaks at 531.4 eV and 533.0 eV were assigned to the C=O and C–OH, respectively. For the O1s peaks of the PEG/PDAM-3, the participation of PEG induced the shifts of two peaks of C=O (532.1) and C–OH (532.7) for 0.3 eV and 0.7 eV, respectively, which implied that the catechol of the PDAM combined with the O and H atoms of PEG to form hydrogen bonds [43]. The XPS survey spectra curves of the PDAMs and PEG/PDAM-3 are shown in Figure 13. It was clear that three peaks could be found in the spectrum of the PDAM, and the spectrum of PEG/PDAM-3 had two high-intensity sharp peaks and one low-intensity peak, which belonged to C, N, and O. Furthermore, the N1s peak in the PEG/PDAM-3 spectrum was smaller compared with that in the spectrum of the PDAMs because the PEG did not contain N and, therefore, the content of N decreased in the composite of PEG/PDAM-3.

### 3.9. Comparison of PEG//PDAM-3 with Other Composite Materials

The latent heat of PEG/PDAM-3 was compared with other form-stable phase change materials, which were also developed by physical adsorption through compounding PEG with supporting materials, and the results are shown in Table 2. It was clear that the thermal storage capacity of PEG/PDAM-3 was favourable and competitive, though the melting and freezing latent heats of PEG/PDAM-3 were not the highest. Moreover, PEG/PDAM-3 had excellent thermal stability and reliability, and the synthetic method of producing the PDAMs and PEG/PDAM-3 had a series of advantages, such as easy operation, mild conditions, and environmental friendliness. Therefore, PEG/PDAM-3 has the greatest potential in practical applications for thermal energy storage.

## 4. Conclusions

In this work, a novel high-efficient form-stable phase change material of PEG/PDAM was developed using a simple and environmentally friendly method. A series of tests, including SEM, TEM, XRD, DSC, XPS, BET, FTIR, and TGA, were carried out to study the properties of PEG/PDAMs. The results indicated that PEG adsorbed on the surface of PDAMs very well, and the interaction mechanisms between the PEG molecules and the PDAMs were hydrogen bonds linkages. The phase change property study results demonstrated that the PEG/PDAMs had high latent heat storage capacity, favourable thermal stability, and reliability, which enables it to have great potential in practical applications for thermal energy storage. Furthermore, because PDAMs have strong adsorption capacity for various organic materials, it can be used as a matrix for other organic materials in preparation of form-stable phase change materials.

## Figures and Tables

**Table 1:** Phase change temperatures and latent heat values of the PEG and PEG/PDAM composites ^a^.

Samples	*T*_mo_ (°C)	*T*_mp_ (°C)	*T*_me_ (°C)	Δ*H*_m_ (J/g)	*T*_co_ (°C)	*T*_cp_ (°C)	*T*_ce_ (°C)	Δ*H*_c_ (J/g)
PEG/PDAM-1	56.00 ± 0.00	60.9 ± 0.15	65.45 ± 1.05	82.57 ± 4.27	23.70 ± 1.40	28.05 ± 1.05	34.75 ± 0.05	66.10 ± 4.16
PEG/PDAM-2	54.55 ± 0.95	62.00 ± 0.00	66.10 ± 0.70	108.65 ± 1.85	25.10 ± 1.10	29.80 ± 0.80	34.50 ± 2.00	91.17 ± 2.44
PEG/PDAM-3	57.35 ± 0.05	62.20 ± 0.00	68.00 ± 0.50	133.20 ± 2.50	26.80 ± 0.80	31.10 ± 0.60	35.90 ± 0.80	107.55 ± 4.45
PEG	59.05 ± 0.15	63.30 ± 0.60	66.05 ± 0.85	227.40 ± 2.00	32.30 ± 0.10	35.95 ± 0.35	40.25 ± 0.65	207.10 ± 0.00

^a^*T*_mo,_*T*_mp_, and *T*_me_ are the onset, the peak, and the end of the melting phase change temperature, respectively. *T*_co_, *T*_cp_, and *T*_ce_ are the onset, the peak, and the end of the freezing phase change temperature, respectively. Δ*H*_m_ and Δ*H*_c_ are the latent heat values in the melting process and in the freezing process, respectively.

**Table 2:** Comparison of PEG/PDAM-3 with other composite materials.

Samples	Melting		Freezing		References
	ΔHm (J/g)	Tm_p_ (°C)	ΔHc (J/g)	Tc_p_ (°C)	
PEG/PDAM-3	133.20 ± 2.50	62.20 ± 0.00	107.55 ± 4.45	31.10 ± 0.60	Present study
PEG/AC ^a^	81.30	49.00	72.80	27.80	[48]
PEG/Dop-SF-3 ^b^	73.80	53.00	69.10	44.80	[43]
PEG/RMS ^c^	129.60	57.22	118.30	39.02	[49]
PEG/SiO_2_^d^	151.80	58.09	141.00	42.34	[10]
PEG/ZSM-5c ^e^	76.40	60.50	64.30	44.90	[35]

^a^ PEG/AC: 80 wt.% of polyethylene glycol/active carbon. ^b^ PEG/Dop-SF-3: 70 wt.% of polyethylene glycol/dopamine functionalized silica fume. ^c^ PEG/RMS: 80 wt.% of polyethylene glycol/radial mesoporous silica. ^d^ PEG/SiO_2_: 79.3 wt.% of polyethylene glycol/SiO_2_. ^e^ PEG/ZSM-5c: 50 wt.% of polyethylene glycol/mesoporous ZSM-5.
